# Characterization of a Species E Adenovirus Vector as a Zika virus vaccine

**DOI:** 10.1038/s41598-020-60238-5

**Published:** 2020-02-27

**Authors:** Brianna L. Bullard, Brigette N. Corder, David N. Gordon, Theodore C. Pierson, Eric A. Weaver

**Affiliations:** 1 School of Biological Sciences, Nebraska Center for Virology, University of Nebraska, Lincoln, USA; 2 Laboratory of Viral Diseases, National Institute of Allergy and Infectious Diseases, NIH, Bethesda, Maryland USA

**Keywords:** Infectious diseases, Vaccines

## Abstract

The development of a safe and efficacious Zika virus (ZIKV) vaccine remains a global health priority. In our previous work, we developed an Adenovirus vectored ZIKV vaccine using a low-seroprevalent human Adenovirus type 4 (Ad4-prM-E) and compared it to an Ad5 vector (Ad5-prM-E). We found that vaccination with Ad4-prM-E leads to the development of a strong anti-ZIKV T-cell response without eliciting significant anti-ZIKV antibodies, while vaccination with Ad5-prM-E leads to the development of both anti-ZIKV antibody and T-cell responses in C57BL/6 mice. However, both vectors conferred protection against ZIKV infection in a lethal challenge model. Here we continued to characterize the T-cell biased immune response observed in Ad4 immunized mice. Vaccination of BALB/c mice resulted in immune correlates similar to C57BL/6 mice, confirming that this response is not mouse strain-specific. Vaccination with an Ad4 expressing an influenza hemagglutinin (HA) protein resulted in anti-HA T-cell responses without the development of significant anti-HA antibodies, indicating this unique response is specific to the Ad4 serotype rather than the transgene expressed. Co-administration of a UV inactivated Ad4 vector with the Ad5-prM-E vaccine led to a significant reduction in anti-ZIKV antibody development suggesting that this serotype-specific immune profile is capsid-dependent. These results highlight the serotype-specific immune profiles elicited by different Adenovirus vector types and emphasize the importance of continued characterization of these alternative Ad serotypes.

## Introduction

Since the 2015/16 Zika virus (ZIKV) outbreak in Brazil, there has been continued progress towards the development of a safe and efficacious vaccine. Many vaccine platforms have been explored in pre-clinical studies, such as live attenuated^1–3^, inactivated^4,5^, subunit^6,7^, DNA^8–10^, mRNA^11,12^, and viral vectors^13,14^. Phase I clinical trials have been completed using a purified inactivated virus^5^ and DNA plasmids expressing the ZIKV prM-E genes^10,15^ and have shown mild to moderate adverse events with promising seroconversion rates^5,10,15^. In addition, multiple other phase I clinical trials are currently recruiting, including two viral vectored vaccines using a measles virus vector (MV-ZIKV; NTC04033068) or a chimpanzee adenovirus vector (ChAdOx1^16^; NTC04015648).

Adenovirus (Ad) viral vectors have shown efficacy in preclinical and clinical trials against ZIKV, along with many other important infectious diseases such as influenza^17,18^, HIV^19,20^, and Ebola^21–23^. Traditionally, Adenovirus type 5 (Ad5) has been the most commonly used Ad vector. However, high levels of pre-existing immunity to Ad5 has led to the development of alternative Ad vectors based on low-seroprevalent human or animal Adenoviruses^24^. Along with Ad5 vectors^25–27^, vaccines for ZIKV have been developed using Adenovirus type Ad2^28^, Ad26^29,30^, rhesus monkey Ad52^8^, chimpanzee Ad7^31^, and gorilla Ad^32^.

In our previous work, we developed an Adenovirus vectored ZIKV vaccine using a low-seroprevalent human Adenovirus type 4 (Ad4-prM-E) and compared it to an Ad5 vector (Ad5-prM-E)^33^. Both vaccines expressed the ZIKV prM-E gene under a CMV promoter in place of the essential E1 Adenovirus gene, resulting in replication-defective vectors. Ad5-prM-E vaccination induced significant anti-ZIKV antibody and T-cell responses. In contrast, Ad4-prM-E vaccination resulted in a significant anti-ZIKV T-cell response without the development of detectable antibodies. However, both Ad5-prM-E and Ad4-prM-E protected against weight loss and death in a lethal antibody blockade ZIKV challenge using C57BL/6 mice that were treated with a blocking anti-IFNAR1 antibody one day before challenge. The unique immune profile after vaccination with the Ad4-prM-E vaccine warranted further characterization of this vector.

In this study, we continue to characterize the Ad4-prM-E ZIKV vaccine. First, we use a reporter virus particle (RVP) based assay to measure ZIKV binding and neutralizing antibodies after Ad4-prM-E vaccination. Additionally, we evaluate the absence of antibody development after Ad4 vector vaccination in a second mouse strain or with expression of a different transgene. Finally, we assess the role of the Ad4 capsid in the T-cell biased immune response observed in Ad4 immunized mice. These results highlight the different immune profiles elicited by Adenovirus serotypes and stress the need for continued characterization of these alternative Adenovirus.

## Results

### Ad4-prM-E shows similar protein expression to Ad5-prM-E *in vitro*

**Figure 1:**
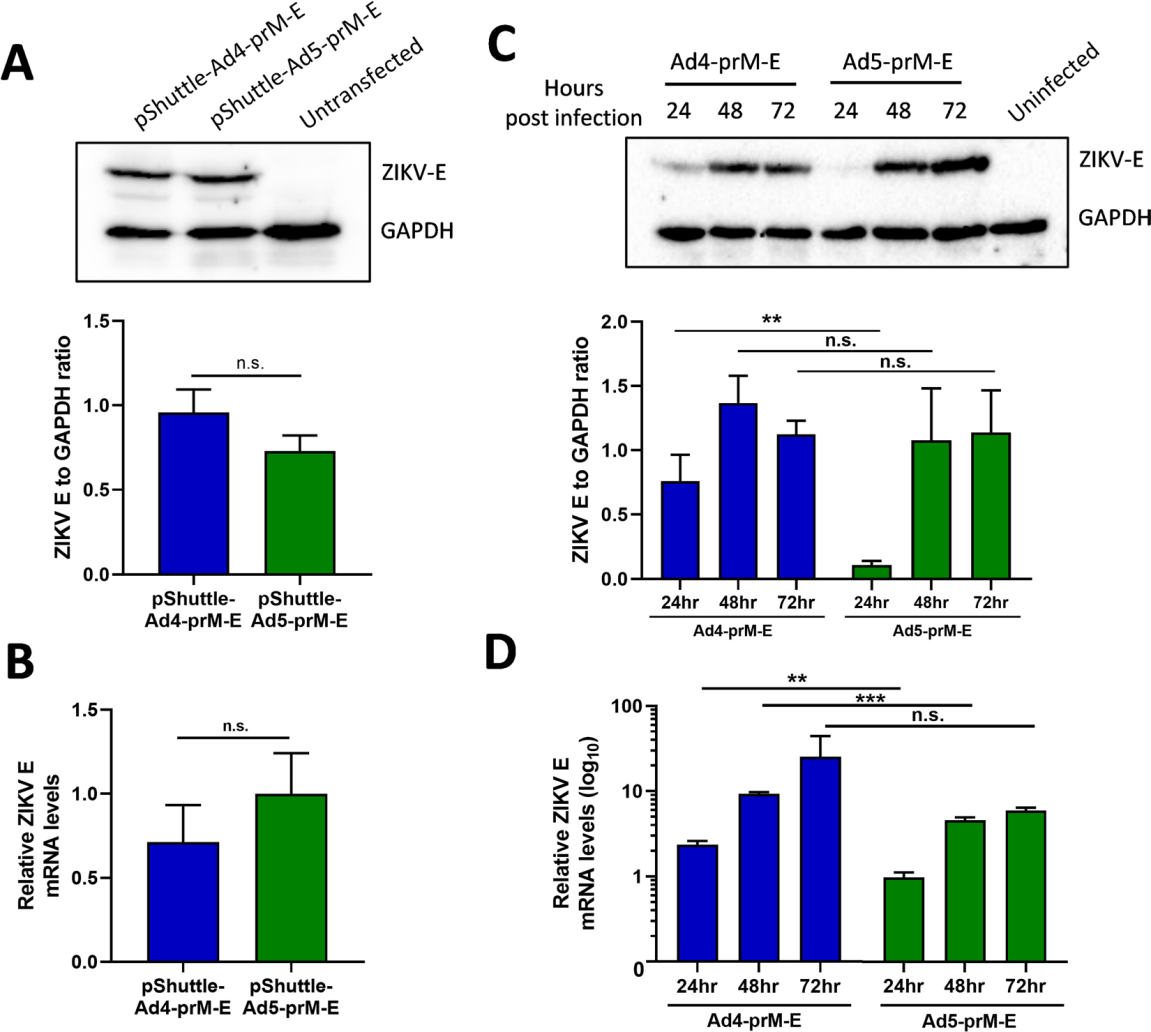
ZIKV E protein expression and mRNA levels *in vitro*. (**A**,**B**) The pShuttle plasmids expressing the ZIKV prM-E which were used to create the recombinant Ad4 and Ad5 vectors were transfected at equal copy number into 293 cells. After 48 hours, the cells were harvested and evaluated for ZIKV E protein expression by western blot. Panel A shows a representative western blot with GAPDH cellular control along with quantification of the ratio of ZIKV E protein expression to GAPDH control (n.s. not significant; two-tailed T-test). In addition, qPCR was used to evaluate ZIKV E mRNA levels post transfection. (**B**) Samples were normalized to β-actin mRNA and represented relative to pShuttle-Ad5-prM-E (n.s. not significant; two-tailed T-test). To evaluate protein expression after infection with the Ad vectors, non-complementary A549 cells were infected at 1,000vp/cell with either Ad4-prM-E or Ad5-prM-E and washed after 1 hour. Cells were harvested at 24, 48, and 72 hours post infection to determine protein expression by western blot as described in panel A. (**C**) ZIKV E mRNA levels at each timepoint were determined with qPCR. (**D**) Samples were normalized to β-actin mRNA and represented relative to Ad5-prM-E infection at 24 hours (*p < 0.05, **p < 0.01, ***p < 0.001; two-tailed T-test). All experiments were repeated in triplicate and data are expressed as the mean with standard deviation.

Vaccination with the Ad4-prM-E and Ad5-prM-E vectors led to the development of anti-ZIKV antibodies in the Ad5-prM-E vaccinated animals but not the Ad4-prM-E. We have previously shown equivalent levels of ZIKV E protein expression *in vitro* after infection of complementing 293 cells^33^, however we wanted to further confirm comparable levels of protein expression *in vitro*. We started by transfecting 293 cells with equal copy numbers of the pShuttle cloner plasmids expressing the ZIKV E protein that was used to create the recombinant Ad4 or Ad5. After 48 hours, both transfections showed equivalent protein expression of the ZIKV envelope (E) protein as measured by western blot (Fig. 1A) along with equivalent ZIKV E mRNA levels (Fig. 1B) confirming equal transgene expression from the pShuttle cloning plasmids. We then wanted to evaluate transduction and expression in non-complementing A549 cells which would more closely mimic conditions of vaccination with a replication-defective Adenovirus. A549 cells were infected with 1,000 virus particles (vp) per cell of either Ad4-prM-E or Ad5-prM-E, washed after 1 hour, and then collected at 24, 48, and 72 hours post infection. Surprisingly, western blotting showed ZIKV E protein expression levels were stronger in the Ad4-prM-E infection as compared to the Ad5-prM-E at 24hrs. However, the protein expression of ZIKV E were equivalent between Ad4-prM-E and Ad5-prM-E by 48 and 72hrs (Fig. 1C). Supporting this, the ZIKV E mRNA levels were higher in Ad4-prM-E infection in the early time points (Fig. 1D). This data indicates that Ad4-prM-E infection might lead to faster transgene expression as compared to Ad5-prM-E but that protein expression equalized by 48 to 72 hours. This supports that the differential immune response seen after vaccination with Ad4-prM-E is likely not a result of reduced transgene expression of this vector.

### Ad4-prM-E does not induce detectable anti-ZIKV antibodies

**Figure 2:**
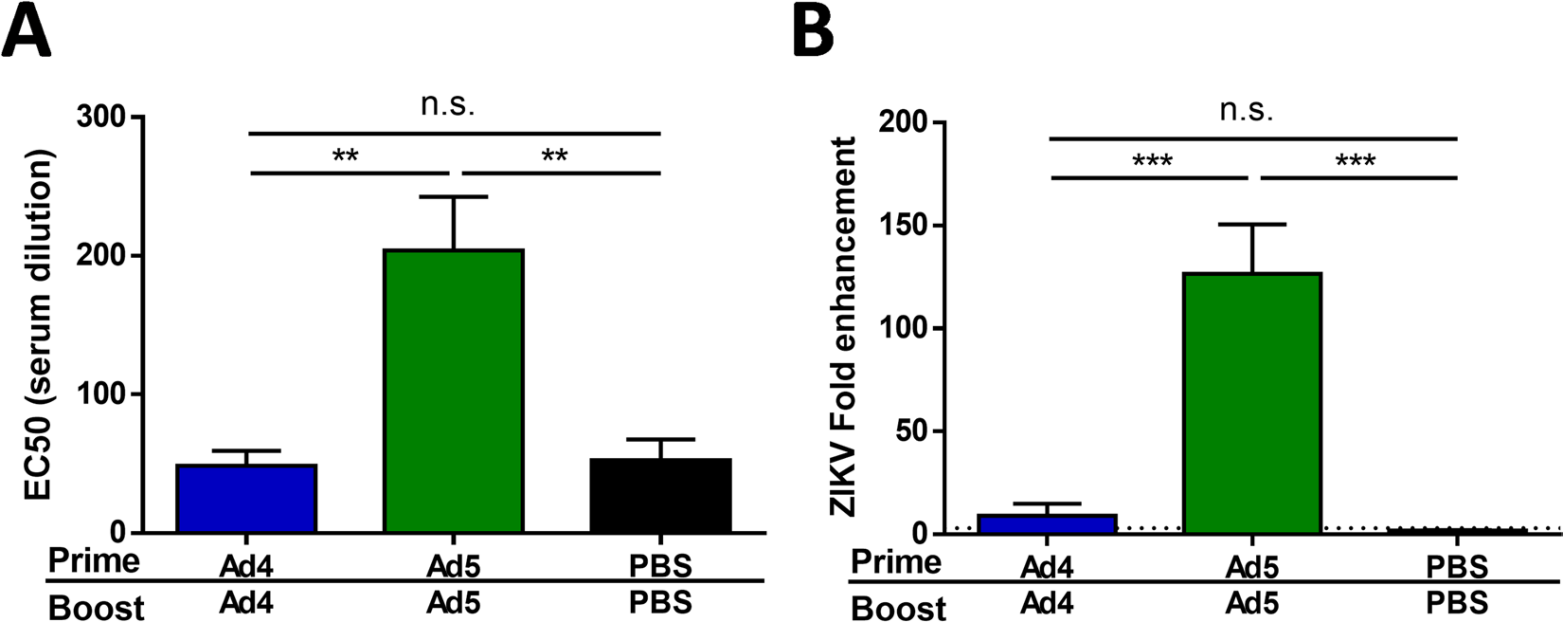
Anti-ZIKV antibody development after vaccination. (**A**,**B**) C57BL/7 mice were vaccinated via the i.m. route with 10^10^ vp of Ad5-prM-E (n = 5), Ad4-prM-E (n = 4), or PBS (n = 5) with a prime boost strategy. Anti-ZIKV antibodies were measured by reporter virus particle (RVP) neutralization assay (**A**), or antibody-dependent enhancement assay. (**B**) The dilution of sera required to inhibit 50% of RVP infection events (EC50) is reported. The limit of detection of these assays was a reciprocal serum dilution of 60, with negative samples reported as half the limit of detection (30). The magnitude of ADE is reported as the largest increase of GFP-expressing K562 cells observed at any serum dilution as compared to the no serum control (expressed as fold-increase over background). The dashed line indicates the limit of detection in this assay (3-fold increase over background). Data are expressed as the mean with standard error (SEM).

**Figure 3:**
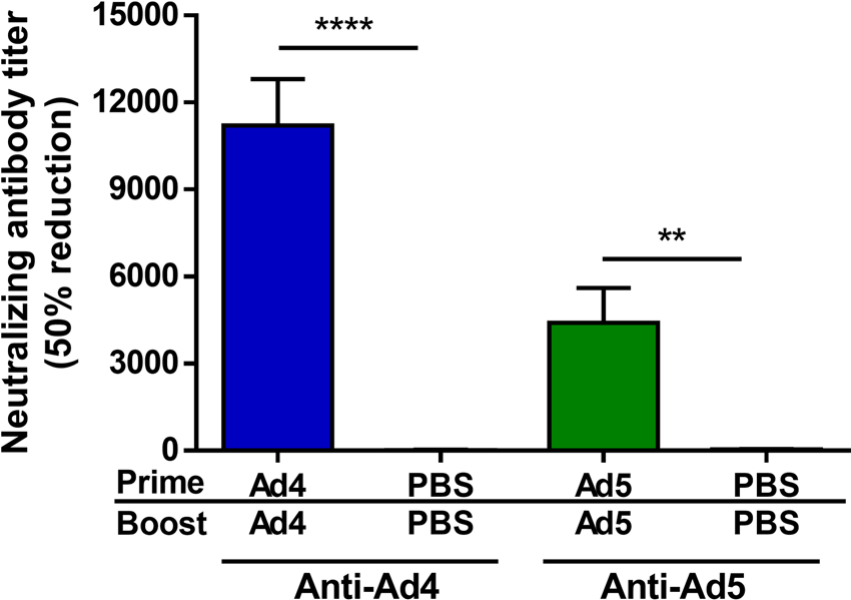
Antibody development against the Adenovirus vectors. C57BL/7 mice (n = 5) were vaccinated via the i.m. route with 10^10^ vp the indicated virus with a prime boost strategy. The antibody response against the Adenovirus vector was measured by neutralization assay. Sera was assayed for its ability to neutralize Ad4 or Ad5 virus expressing GFP-luciferase and the neutralization titer was determined to be the titer at which there was 50% neutralization of the luciferase activity (**p < 0.01, ****p < 0.0001; one-way ANOVA). Data are expressed as the mean with standard error (SEM).

To evaluate the anti-ZIKV immune response after vaccination, C57BL/6 mice were vaccinated with 10^10^ virus particles (vp) of Ad4-prM-E or Ad5-prM-E intramuscularly, boosted 6 weeks later with the same vaccine and dose, and sacrificed 2 weeks after boosting. Our previous work used ELISA and plaque reduction neutralization assays to evaluate the anti-ZIKV antibody response^33^. To further assess antibody development after vaccination, we utilized a sensitive and quantitative reporter virus particle (RVP) neutralization assay used in the preclinical and clinical evaluation of other vaccine candidates^15^. RVP assays revealed significant levels of anti-ZIKV neutralizing antibodies after vaccination with Ad5-prM-E; whereas, no detectable neutralizing antibodies were observed after vaccination with Ad4-prM-E (Fig. 2A). Neutralization assays may have limited sensitivity due to a requirement for antibodies to bind individual virions in sufficient numbers to block infection. Conversely, antibodies that bind the virion with stoichiometry insufficient for neutralization may promote infection of cells expressing Fc-receptors via a process referred to as antibody dependent enhancement of infection (ADE). ADE assays using FcγRII-expressing K562 cells were performed to detect the presence of non-neutralizing anti-ZIKV antibodies (Fig. 2B). While sera from Ad5-prM-E vaccinated mice robustly enhanced ZIKV RVP infection, this was not observed in assays with sera from Ad4-prM-E vaccinated mice. These findings indicate Ad4-prM-E-vaccination elicits very low levels of ZIKV binding or neutralizing antibodies. In contrast, both Ad4-prM-E and Ad5-prM-E vaccination led to a significant anti-Adenovirus neutralizing antibody response (Fig. 3). Thus, although Ad4-prM-E vaccinated mice do not mount an anti-ZIKV antibody response, the mice do form functionally neutralizing antibodies to the Adenovirus vector itself.

### Vaccination of BALB/c mice does not result in detectable anti-ZIKV antibodies

**Figure 4:**
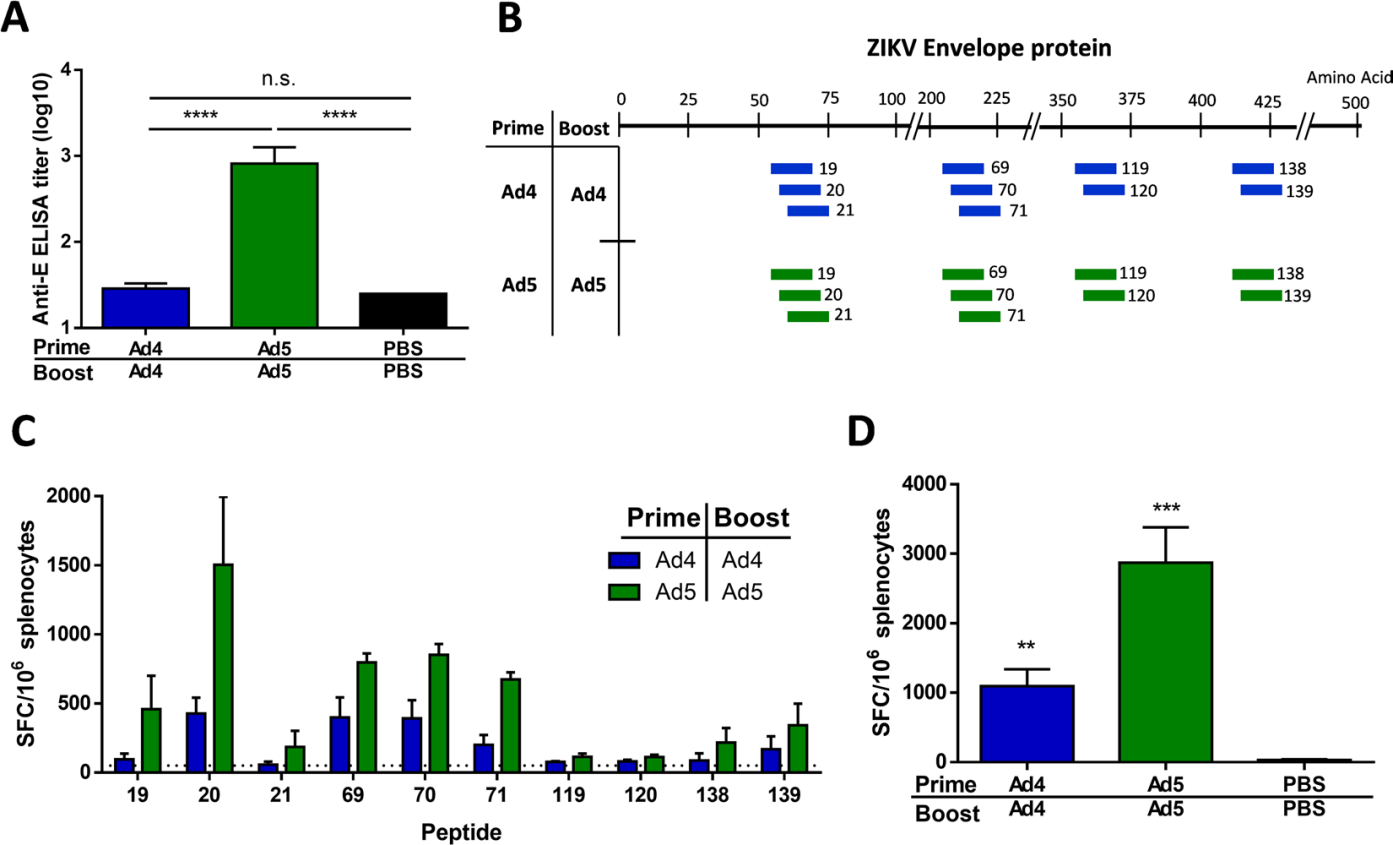
Antibody response and T-cell epitope mapping in BALB/c mice after vaccination. (**A–D**) BALB/c mice (n = 5) were vaccinated with 10^10^ vp of Ad4-prM-E, Ad5-prM-E, or PBS via the i.m. route with a prime boost strategy. ZIKV E protein specific antibodies were measured using an ELISA. (**A**) T-cell epitopes induced against the ZIKV envelope protein was mapped using a 164-peptide array consisting of 15-mers with 13 amino acid overlap. Peptides were considered positive if the response was greater than 50 spot forming cells (SFC) per million. The positive peptides induced by both vaccination strategies can be seen mapped back to the 504 amino acid ZIKV envelope protein in (**B**). The response against each positive peptide is shown with the dashed line at the 50 SFC/10^6^ cutoff (**C**). The total T-cell response against the ZIKV E protein was also measured (**D**) (n.s. not significant, ***p < 0.01, ***p < 0.001; one-way ANOVA). Data are expressed as the mean with standard error (SEM).

C57BL/6 mice are known to have a Th_1_ dominant immune response^34^. Therefore, to ensure that the lack of humoral immune response observed after vaccination with Ad4-prM-E is not mouse strain-specific, we vaccinated BALB/c mice which have a Th_2_ dominant immune response^35^. Mouse strain-dependent effects have been previously observed after vaccination with a DNA ZIKV vaccine^36^ further justifying investigation in a second mouse strain. BALB/c mice were vaccinated with 10^10^ vp of either Ad4-prM-E or Ad5-prM-E, boosted 3 weeks later with the same vaccine and dose, and then sacrificed 2 weeks after boosting. ZIKV envelope specific antibodies were measured using an ELISA in order to detect binding antibodies regardless of their ability to functionally neutralize (Fig. 4A). Similar to results of studies in the C57BL/6 model, vaccination of BALB/c mice with Ad5-prM-E resulted in significant anti-ZIKV antibody development. However, there were no detectable anti-ZIKV antibodies after vaccination with Ad4-prM-E, confirming that the absence of antibody development is independent of the mouse strain.

### Mapping of T-cell epitopes in BALB/c mice

We went on to map the anti-ZIKV envelope T-cell epitope response in BALB/c mice after vaccination using an interferon-γ enzyme-linked immunospot assay (ELISpot) with an overlapping peptide array spanning the entire ZIKV E protein. Peptides that induced spot forming cells (SFC) responses greater than 50 per million splenocytes were considered positive. Both Ad5-prM-E and Ad4-prM-E vaccination induced T-cell responses against the same peptides in the envelope protein (Fig. 4B,C). In addition, both vectors induced a significant total T-cell response against the ZIKV E protein (Fig. 4D). Previous work has shown that, in C57BL/6 mice, the immunodominant epitope in the ZIKV envelope protein is E_4–12_^33,37–39^. In BALB/c mice, a different immunodominance pattern was observed as compared to the C57BL/6 mice. In C57BL/6 mice, the E_4–12_ region showed clear immunodominance with over 5,000 and 7,000 SFC/million after vaccination with the Ad4-prM-E and Ad5-prM-E ZIKV vaccines, respectively^33^. However, in BALB/c mice, peptides 19, 20, and 21 encompassing the region E_55–75_ and peptides 69, 70, and 72 encompassing the region E_205–225_ show the strongest T-cell response after vaccination with both vectors (Fig. 4C). These responses reached levels of approximately 1,500 and 850 SFC/million, respectively.

### Adenovirus 4 expressing influenza hemagglutinin results in no detectable antibodies but significant T-cell responses

**Figure 5:**
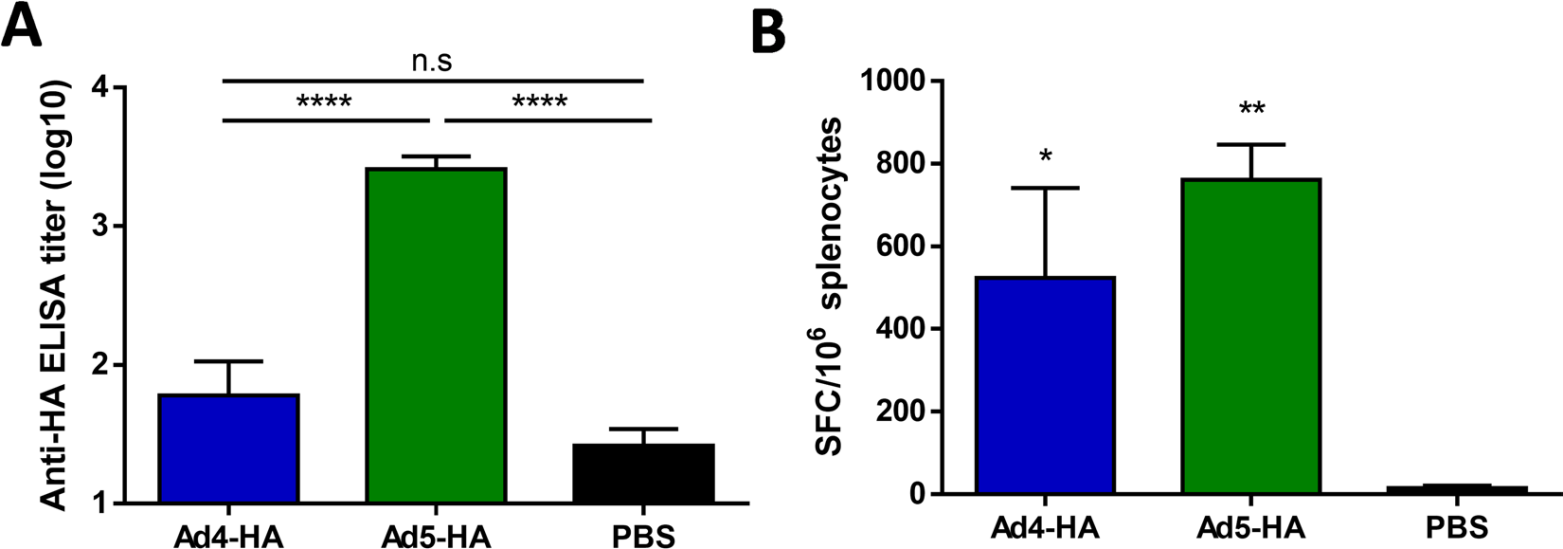
Antibody and T-cell response after vaccination with Adenovirus 4 or 5 expressing an influenza hemagglutinin transgene. (**A**,**B**) C57BL/6 mice (n = 5) were vaccinated with an Adenovirus 4 or 5 vector expressing a consensus hemagglutinin H1 influenza gene. Hemagglutinin (HA) specific antibodies to the H1N1 virus PR/8/34 were measured using an ELISA (**A**) (n.s. not significant, ****p < 0.0001; one-way ANOVA). The total T-cell response against the HA of A/PR/8/34 was determined with pooled peptides from a peptide array covering the entire HA and are reported as spot forming cells (SFC) per million (**B**) (*p < 0.05, **p < 0.01, one-way ANOVA). Data are expressed as the mean with standard error (SEM).

Next, we wanted to confirm that the lack of humoral immune response after Ad4-prM-E vaccination is not transgene-specific. Therefore, we vaccinated C57BL/6 mice with 10^10^ vp of Adenovirus 4 and 5 vectors expressing a consensus H1 influenza hemagglutinin (HA) and sacrificed the mice 2 weeks later in order to evaluate the anti-HA antibody and T-cell response. The influenza HA protein is known to induce a strong humoral immune response when expressed in a viral vector making this an ideal transgene to study induction of antibody responses^17,40^. In support of this, we found that Ad5-HA vaccination resulted in significant anti-HA antibody response as measured by ELISA (Fig. 5A). In contrast, Ad4-HA vaccination resulted in no significant anti-HA antibody development. However, both Ad vaccines were able to induce significant anti-HA total T-cell responses (Fig. 5B). This indicates that a similar immune profile of T-cell development with the absence of antibodies is observed after Ad4 vector vaccination, regardless of the transgene expressed.

### Co-administration of Ad5-prME with Ad4-GFP reduces anti-ZIKV antibody development

**Figure 6:**
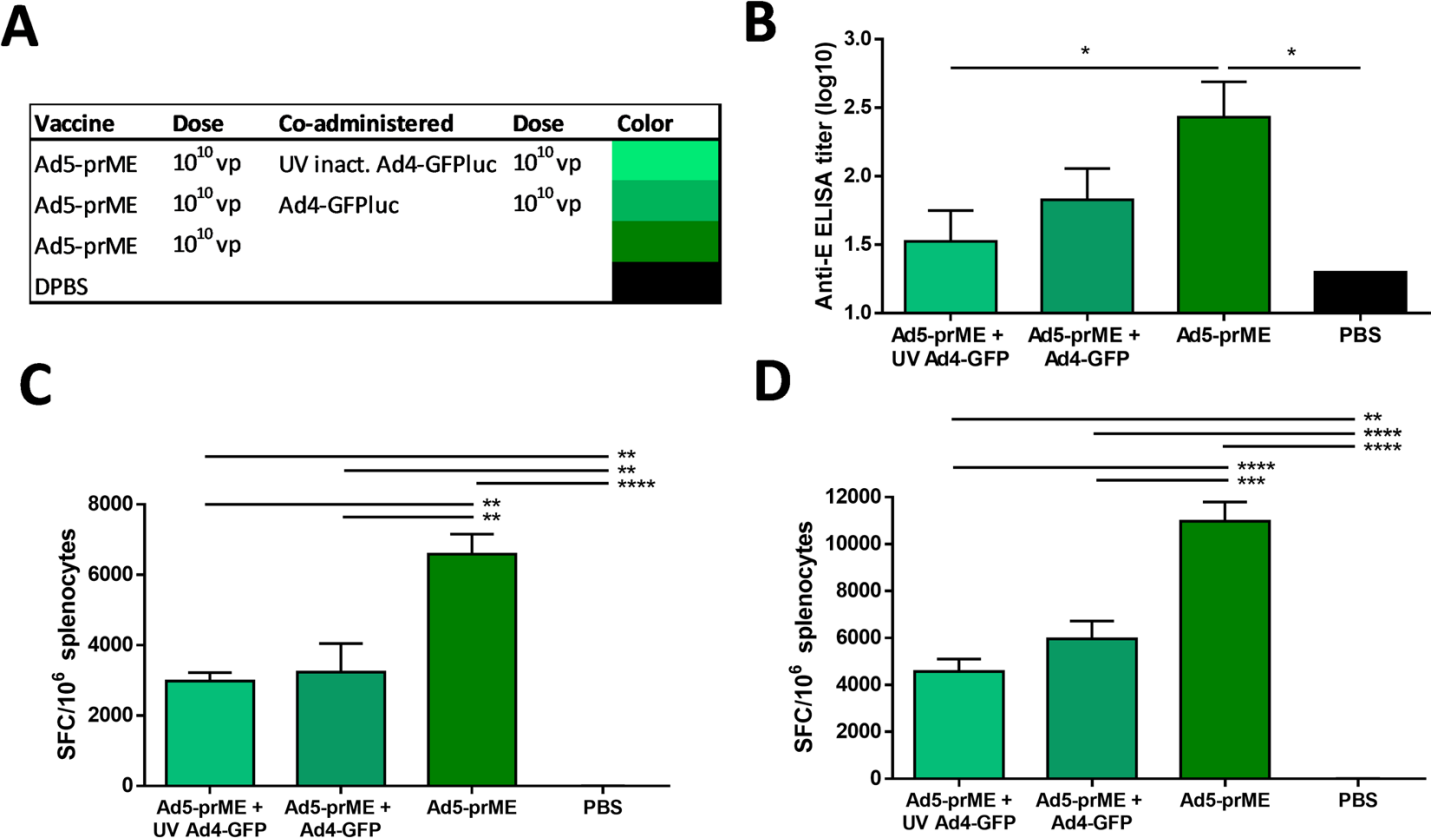
Co-administration with an Ad4-GFP vector reduces antibody development from vaccination with Ad5-prM-E. (**A–D**) C57BL/6 mice (n = 4) were vaccinated via the i.m. route with the indicated vaccine(s) and dose and sacrificed 2 weeks later (**A**). ZIKV E protein specific antibodies were measured with an ELISA (**B**). The T-cell response against the previously described immunodominant CD8 + epitope for the ZIKV E protein in C57BL/6 mice was measured^33,37^ (**C**) along with the total T-cell response to the ZIKV E protein (**D**) and reported as spot-forming cells (SFC) per million) (*p < 0.05, **p < 0.01, ****p < 0.0001; one-way ANOVA). Data are expressed as the mean with standard error (SEM).

To determine if the T-cell biased immune response seen after vaccination with Ad4 is capsid-dependent, we performed a co-administration study. C57BL/6 mice were vaccinated with 10^10^ vp of Ad5-prM-E alone or co-administered with 10^10^ vp of Ad4 expressing GFP-Luciferase (Ad4-GFPluc) or with 10^10^ vp of UV inactivated Ad4-GFPluc (Fig. 6A) and sacrificed 2 weeks later. UV inactivation was performed by UVc radiation and confirmed by an infectivity assay in 293 cells (data not shown). The UV inactivation of the Ad4-GFPluc ensures that expression of Ad genes will not play a role in immune modulation. We have shown that Ad5-prM-E vaccination leads to development of significant anti-ZIKV antibodies. However, when Ad5-prM-E was co-administered with Ad4, anti-ZIKV antibody titers decrease significantly (Fig. 6B). The T-cell response against the C57BL/6 immunodominant CD8+ cytotoxic T lymphocyte (CTL) epitope (Fig. 6C) and the total T-cell response (Fig. 6D) remained significant for all Ad5-prM-E vaccinated mice. The CTL and overall T cell responses in the Ad5 co-administered with UV-inactivated Ad4 and Ad4-GFP were significantly lower than the Ad5 responses alone (p = ≤0.01) **(**Fig. 6C,D**)**. This indicates that the biased immune response during Ad4 vector vaccination is likely capsid-dependent.

## Discussion

Development of a safe Zika virus (ZIKV) vaccine remains a global health priority^41^. Our previous work has shown that vaccination with an Adenovirus 4 vector expressing the Zika virus (ZIKV) prM-E genes leads to the development of a strong anti-ZIKV T-cell response without the development of anti-ZIKV antibodies^33^. This is in contrast to the Ad5-prM-E vaccine which induced both anti-ZIKV T-cell and antibody responses. However, both vectors conferred protection against ZIKV infection in a lethal antibody blockade challenge model. Here, we continued to characterize the Adenovirus 4 vector.

RVP neutralization and enhancement assays failed to detect neutralizing or binding anti-ZIKV antibodies after Ad4-prM-E vaccination. Vaccination of BALB/c mice with Ad4-prM-E showed similar results to C57BL/6 mice, indicating that this is not a mouse strain-specific response. In addition, expression of an influenza hemagglutinin gene by Ad4 and Ad5 showed that, while Ad5-HA vaccination induced significant anti-HA antibody and T-cell responses, Ad4-HA vaccination only resulted in significant T-cell responses with no detectable anti-HA antibodies. These results indicate that the unique immune response after Ad4 vaccination is specific to the Ad4 serotype itself and not a mouse strain or transgene specific response.

Additionally, we found that co-administration of Ad5-prM-E with a UV inactivated Ad4-GFPluc significantly reduced the anti-ZIKV antibodies as compared to the Ad5-prM-E alone. This result supports that the serotype specific immune response after Ad4 vaccination is likely specific to the capsid proteins. However, UV inactivated Adenovirus can still transduce cells^42^ so this does not rule out other internal structural proteins packaged with the virion. Further experiments are needed to determine the specific structural protein responsible for the observed differences in the antibody development.

Our results here support the findings of another study utilizing an Ad4 vector expressing LacZ. This study found that enhanced innate immunogenicity after Ad4 vector administration was capsid-dependent and interfered with transgene specific antibody responses without diminishing the T-cell response^43^. This study found stronger stimulation of multiple cytokines, including IFN-β, IL-6, and TNF-α, after administration of the Ad4 vector as compared to the Ad5 vector. Another study characterizing a low seroprevelent chimpanzee Adenovirus type 68, found significantly increased type 1 interferons after vector administration as compared to Ad5 which lead to a reduction of transgene-specific antibody responses while still inducing a significant T-cell response^44^. However, in our previous study^33^, vaccination of interferon receptor knock out mice (IFNAR-/-) with the Ad4 and Ad5 vector ZIKV vaccines still did not result in a significant anti-ZIKV antibody response. This indicates that there are likely multiple cytokines, chemokines, or immune modulators playing a role in the Ad4 vector immune response.

In general, Ad4-prM-E vaccination induces lower, but still highly significant, levels of T cell immunity as compared to Ad5-prM-E vaccination^33^. Interestingly, when co-administered with a UV-inactivated Ad4-GFPluc, there is a statistically significant reduction in CTL and overall T cell responses, as compared to Ad5-prM-E alone. Therefore, Ad4 capsid proteins alone may be responsible for down regulating both cellular and humoral immune responses. Further study into this capsid-dependent altered immunogenicity could provide beneficial insight into Adenovirus self-adjuvanting properties and the effect this has on downstream adaptive immunity.

Our continued characterization of the two Adenovirus vectored ZIKV vaccines has highlighted the difference in serotype-specific immunity induced by Adenovirus vectors. Recently, many vaccines utilizing alternative Adenovirus vectors have been developed for a multitude of infectious diseases. However, our research and others has shown that different Ad serotypes evoke unique, serotype-specific immune responses^45–47^. Therefore, low seroprevalence should not be the only consideration when selecting an Adenovirus serotype. Further characterization of these alternative Ad vectors is necessary to ensure selection of the serotype with the desired immune profile.

## Materials and Methods

### Ethics statement

Female C57BL/6J or BALB/c mice ages 6–8 weeks were purchased from Jackson Laboratory. Mice were housed in the Life Sciences Annex building on the University of Nebraska – Lincoln (UNL) campus under the Association for Assessment and Accreditation of Laboratory Animal Care International (AAALAC) guidelines. The protocols were approved by the UNL Institutional Animal Care and Use Committee (IACUC) (Project ID 1448: Viral Vectored Flavivirus Vaccines). All animal experiments were carried out according to the provisions of the Animal Welfare Act, PHS Animal Welfare Policy, the principles of the NIH Guide for the Care and Use of Laboratory Animals, and the policies and procedures of UNL. All immunizations and bleeds were performed under ketamine and xylazine or isoflurane induced anesthesia.

### Recombinant adenovirus construction

Replication-defective Adenovirus 4 and 5 vectors expressing either the ZIKV prM-E gene, the influenza H1 consensus hemagglutinin gene, or GFP-Luciferase were constructed as previously described^18,33,48,49^. The prM-E gene (amino acid 126–794 of the polypeptide) from the PRVABC59 strain was codon-optimized for human gene expression and a VSV G signal was added to the N-terminus of the protein^33^. The influenza H1 consensus hemagglutinin gene was designed as previously described^18,49^. All genes were cloned into an expression cassette containing a CMV promoter and SV40 Poly A signal and then recombined into the E1 region of either the Adenovirus type 4 or type 5 genome.

Recombinant Adenovirus 5 was constructed using the AdEasy Adenoviral Vector System (Agilent). Briefly, the transgene was cloned into a shuttle plasmid containing a CMV promoter and SV40 polyA signal. This shuttle plasmid was linearized and cotransformed into BJ5183 cells with the Adenovirus 5 genome in order to undergo homologous recombination. The recombination inserts the transgene expression cassette into the E1 region of the Adenovirus genome, creating a replication-defective vector. This recombinant Adenovirus genome was linearized and transfected into 293 cells using Polyfect Transfection reagent (Qiagen). Virus rescue was observed via plaque formation. Cells were then harvested, virus released by 3 freeze thaws cycles, and amplified by sequential passage in 293 cells until final amplification in Corning 10-cell stack (~6300 cm^2^). Final virus stocks were purified by 2 sequential CsCl ultracentrifuge gradients, desalted using Econo-Pac 10DG Desalting Columns (Bio-Rad), and stored at −80 °C in Ad-tris buffer.

Recombinant Adenovirus 4 was constructed as previously described^33,48^. Briefly, the complete Adenovirus type 4 genome was cloned into a single low copy plasmid. A shuttle plasmid to replace the E1 region was created using overlapping PCR products. The expression cassette used for recombinant Ad5 construction that contains the transgene under a CMV promoter and PolyA was fused to an Frt-Zeo-Frt fragment to aid in selection of recombinant colonies. The transgene was sequence confirmed and then cloned into the pAd4 shuttle plasmid. This shuttle plasmid was linearized and cotransformed into BJ5183 cells with the Adenovirus 5 genome in order to undergo homologous recombination. This recombinant Ad4 genome was rescued and purified as described above for Ad5 recombinants.

### Western blotting

Cells were pelleted, denatured using Laemmli buffer plus 2-mercaptoethanol, and incubated at 100 °C for 10 minutes. Samples were then passed through a QIAshredder (Qiagen) and run on a 12.5% SDS-PAGE gel. Protein was transferred to a nitrocellulose membrane, blocked for 30 minutes with 5% milk in TBST, and then incubated overnight with mouse anti-ZIKV E protein antibody (mAb-0302156; BioFront Technologies) at 1:5000 and mouse anti-GAPDH (sc47724; Santa Cruz Biotechnology, Inc.) at 1:2000 in TBST 1% milk. The membrane was then wash 3x with TBST and incubated for 30 minutes at room temperature with goat anti-mouse-HRP conjugated antibody (Millipore Sigma) at 1:2000 in TBST 1% milk. After 3 wash in TBST, the membrane was developed with SuperSignal West Pico Chemiluminescent Substrate (Thermo Scientific).

### qPCR for mRNA levels

Total RNA was extracted from cells using RNeasy Plus Mini kit (Qiagen). Next, mRNA was converted into cDNA using ProtoScript First Strand cDNA Synthesis Kit (New England Biolabs) with Oligo-dT primers. Real time PCR was performed using PowerUp SYBR Green Master Mix (Applied Biosystems) and run on the QuantStudio 3 Real-Time PCR System (Applied Biosystems) using the following conditions: 50 °C for 2 min, 95 °C for 2 min, 40 cycles of 95 °C for 15 s and 60 °C for 1 min, followed by a melt curve. Samples were run with ZIKV E primers (5′-GAGAAGTCCAGGCCTGTTCT-3′ and 5′-GGAGTACCGGATCATGCTGA-3′) and normalized to levels of β-actin mRNA using primers (5′-CCAACCGCGAGAAGATGA-3′ and 5′-GGATAGCACAGCCTGGATG-3′).

### Vaccination and sample collection

All immunizations were performed intramuscularly with a 27-gauge needle into both quadriceps in two 25 µl injections. At sacrifice, all animals were terminally bled via a cardiac puncture and spleens were harvested for T-cell immune assays. Sera was isolated from whole blood with a BD Microtainer Blood Collection Tube (Becton Dickinson) and used for further ELISA and neutralization tests. Mice splenocytes were isolated using a 40 μm Nylon cell strainer (BD Labware), red blood cells were lysed using ACK lysis buffer, and the splenocytes were resuspended in cRPMI at a concentration of 10^6^ splenocytes/mL for use in ELISpot assays.

### ELISA

Immunolon 4 HBX microtiter 96-well strips (VWR) were coated with 150 ng per well of recombinant ZIKV E protein (Cat #:MBS596088; MyBioSource) or recombinant H1 hemagglutinin of A/Puerto Rico/8/1934 (H1N1) (NR-19240, BEI) in bicarbonate/carbonate coating buffer overnight at 4 °C. The plates were blocked with 2% BSA in PBS for 2 hours at room temperature (RT). Sera was serially diluted in 1% BSA in PBS and incubated for 2 hours at RT. The plates were washed 6X with PBST and incubated with goat anti-mouse-HRP antibody (1:5000; Thermo Fisher) in 1% BSA in PBS for 1 hour at RT. After washing 4X with PBST and 2X with PBS, the plate was developed with 1-Step Ultra TMB-ELISA (Thermo Fisher) and the reaction was stopped with 2 M sulfuric acid. The OD450 was detected using a SpectraMax i3x Multi-Mode microplate reader (Molecular Devices) and the endpoint titer was determined as signal that was two and a half times background values.

### ELISPOT

The ZIKV T-cell epitopes were mapped as previously described^33^ using a peptide array of the ZIKV strain PRVABC59 envelope protein (NR-50553). Briefly, potential immunogenic peptides were identified using a matrix of peptides pools, and the epitopes were confirmed using individual peptides. Ninety-six well polyvinylidene difluoride-backed plates (MultiScreen-IP, Millipore) were coated with 50 μl of anti-mouse IFN-γ mAb AN18 (5 µg/ml; Mabtech) overnight at 4 °C, washed, and then blocked with RPMI at 37 °C for 1 hour. Equal volumes (50 µL) of the single-cell suspension splenocytes (10^6^ splenocytes/mL) and peptide (5ug/mL) were added to the wells in duplicate and incubated overnight at 37 °C with 5% CO_2_. The plates were washed with PBS and incubated with 100 μl of biotinylated anti-mouse IFN-γ mAb (1:1000 dilution; Mabtech) diluted in PBS with 1% FBS for 1 hour at RT. Plates were washed with PBS, incubated with 100 µl of streptavidin-alkaline phosphatase conjugate (1:1000 dilution; Mabtech) diluted in PBS 1% FBS for 1 hours at RT, and washed again with PBS. To develop, 100 µl of BCIP/NBT (Plus) alkaline phosphatase substrate (Thermo Fisher) was added to each well and development was stopped by washing several times in dH_2_O. Spot were counted using an automated ELISpot plate reader (AID iSpot Reader Spectrum) and are expressed as spot-forming cells (SFC) per 10^6^ splenocytes. The total T-cell response to the influenza H1 hemagglutinin was measured using pooled peptides from the peptide array A/Puerto Rico/8/1934 (NR-18973) from BEI resources and an ELISpot performed a described above.

### Reporter virus particle-based neutralization and antibody-dependent enhancement assays

The production of RVPs, neutralization assays, and antibody dependent enhancement assays were performed using previously described methods^50^. Briefly, RVPs were produced by complementation of a self-replicating West Nile virus subgenomic replicon encoding a GFP reporter gene with the C-prM-E structural genes from ZIKV H/PF2013. Neutralization and antibody-dependent enhancement assays were performed by serially diluting heat-inactivated immune sera and incubating with ZIKV RVPs for 1 hour at 37 °C. The resulting immune complexes were added to 5 × 10^6^ Raji-DCSIGNR cells or K562 cells (for neutralization and antibody-dependent enhancement assays, respectively) and incubated at 37 °C for 2 days. Infection was measured by flow cytometry as a function of GFP expressing cells. Neutralization titers were estimated by non-linear regression analysis using GraphPad Prism software.

### Adenovirus neutralization assay

Sera was heat inactivated at 56 °C for 30 min before a serial 2-fold dilution was performed in a 96-well black plate (3603 Corning). Fifty microliters of either Ad4-GFP-Luciferase or Ad5-GFP-Luciferase at a concentration of 1 × 10^8^ Ad-GL vp/mL was then added to the sera dilutions and incubated at 37 °C for 1 hour. Next, 50 µl of A549 cells at 1 × 10^5^ cells/mL was added to each well and the plates were incubated for 20 h at 37 °C and 5% CO_2_ before readout. Cells were treated with 5x lysis buffer and luciferase activity was detected by adding 50 µL of firefly luciferase activity reagent (LAR; Promega). Neutralizing antibody titers were determined as the reciprocal dilution to inhibit 50% of luciferase activity as compared to no serum controls.

### Statistical analysis

GraphPad Prism software was used to analyze all data. Data are expressed as the mean with standard error (SEM). ELISA, Adenovirus neutralization, and T-cell data were analyzed using one-way ANOVA. A p-value <0.05 was considered statistically significant (*p < 0.05; **p < 0.01; ***p < 0.001; ****p < 0.0001).

## Supplementary information

Supplemental Information.
