# Supplementary material for: Characterization of a Species E Adenovirus Vector as a Zika virus vaccine
Source: Sci Rep. 2020 Feb 27;10:3613. doi: 10.1038/s41598-020-60238-5 (PMC7046724; doi:10.1038/s41598-020-60238-5)
Supplement: Supplementary file 1 — Supplemental Information. [file 41598_2020_60238_MOESM1_ESM.pdf]

Characterization of a Species E Adenovirus Vector as a Zika virus vaccine

Brianna L. Bullard, Brigette N. Corder, David N. Gordon, Theodore C. Pierson, and Eric A. Weaver

Supplemental Materials

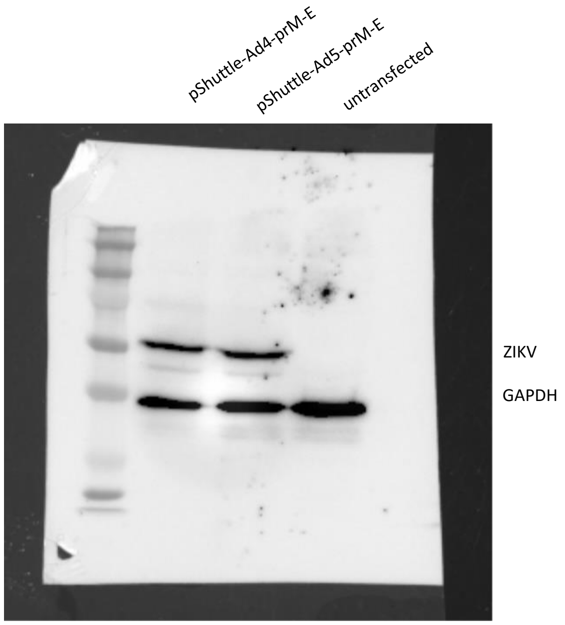

Supp. Fig. 1. Unedited Western Blot with kaleidoscope prestained protein markers in reference to figure 1A.

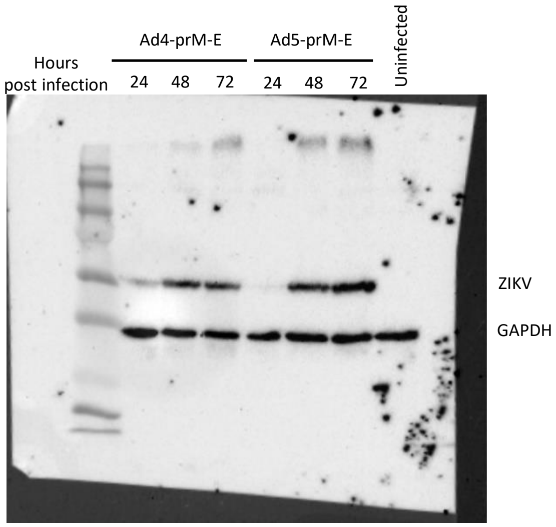

Supp. Fig. 2. Unedited Western Blot with kaleidoscope prestained protein markers in reference to figure 1C.
